# Objective Neural Markers of Identity Familiarization via Passive Exposure and Fast Periodic Visual Stimulation EEG

**DOI:** 10.1523/ENEURO.0328-25.2026

**Published:** 2026-09-08

**Authors:** Tram T. N. Nguyen, Linda Ficco, Ian M. Thornton, Meike Ramon

**Affiliations:** ^1^Department of Cognitive Science, Faculty of Media & Knowledge Sciences, University of Malta, Msida MSD 2080, Malta; ^2^Bern University of Applied Sciences, Applied Face Cognition Laboratory, Bern University of Applied Sciences, Business School, Bern 3005, Switzerland; ^3^Association of Independent Research, Zürich 8037, Switzerland

**Keywords:** CCTV, face-identity learning, fast periodic visual stimulation, implicit identity processing, mugshot images

## Abstract

Despite a wealth of information provided by face perception research, the neural processes by which unfamiliar faces become familiar—particularly via passive exposure—remain poorly understood. Here, we introduce a novel fast periodic visual stimulation electroencephalography (FPVS-EEG) paradigm designed to objectively track neural familiarization. Specifically, our paradigm measures the emergence of implicit face-identity familiarity during brief, passive repetition learning, without requiring explicit recognition or task engagement. The paradigm uses ecologically valid stimuli incorporating image variations characteristic of forensic material. We tested 28 (18 females) healthy human volunteers including neurotypical civilians and law enforcement professionals, all with typical face-processing abilities. Neural responses were measured to a 1 Hz tagged oddball identity embedded within a 6 Hz stream of changing base images, using high-quality mugshot and CCTV-like stimuli. Results showed that neural signatures of implicit familiarity can emerge rapidly within minutes of passive exposure but only when high-quality mugshot images are used. These familiarity-related responses were characterized by concurrent neural suppression and enhancement, likely to reflect the co-occurrence of repetition detection and identity learning processes. The FPVS-EEG approach reported here provides an objective and quantifiable measure of implicit, early face learning that could complement behavioral assessments and inform future research on individual differences and applied contexts.

## Significance Statement

While there is ample research highlighting differences in the processing of unfamiliar and familiar faces, much less is known about how unfamiliar faces become familiar, particularly at the neural level. Here, we developed a novel paradigm based on fast periodic visual stimulation to probe face learning implicitly via passive exposure, without explicit recognition decisions. We demonstrated that neural signals of familiarity emerged within 4 min of exposure to high-quality mugshots, but not degraded CCTV-like images, for individuals with typical face-processing abilities. Our study offers novel neural measures that can objectively track the earliest stages of familiarization, potentially advancing the study of individual differences in face processing and its applied contexts.

## Introduction

### Facial identity processing: familiarity matters

Processing the identity of others is a fundamental cognitive function underpinning human social interaction. In everyday life, faces are commonly categorized as unfamiliar or familiar depending on whether they belong to people we know. Such behavioral familiarity decisions can be made extremely rapidly, within ∼400 ms (Ramon et al., 2011; Besson et al., 2017), only 150–200 ms after the earliest neural signatures of familiarity are detectable (Caharel et al., 2014; Barragan-Jason et al., 2015; Huang et al., 2017). A large body of face-identity processing (FIP) research has demonstrated that, compared with strangers, highly familiar faces are processed faster and with higher accuracy across detection, discrimination, recognition, and identification tasks (Hancock et al., 2000; Johnston and Edmonds, 2009; Ramon and Gobbini, 2018) and are particularly resilient to substantial variations in visual input, including changes in image quality, viewing angle, age, cosmetic modifications, and within-person variability (Bruce and Young, 1986; Jenkins et al., 2011). Importantly, this familiarity advantage is not limited to explicit recognition tasks but is also observed in perceptual face-matching paradigms (Bruce et al., 1999, 2001; Burton et al., 1999; Megreya and Burton, 2008; Megreya et al., 2013), indicating that familiarity enhances perceptual identity processing itself rather than reflecting memory alone.

While there is extensive literature on how our brain represents (often highly) familiar faces relative to faces never seen before, the dominant focus on familiarity as simple dichotomies neglects the wide range of familiarity encountered in everyday life, from fleeting encounters to personally known or famous individuals (Natu and O’Toole, 2011; Kramer et al., 2018b). Kovács (2020) proposed a hierarchical model in which familiarity develops from an initial stage of visual familiarity arising through repeated exposure and supporting the earliest extraction of identity-specific structure, toward progressively richer representations incorporating contextual associations and detailed person knowledge. Yet, much less is known about how the neural representation of a face emerges and changes as a person becomes increasingly familiar (cf. Popova and Wiese, 2022; Wiese et al., 2024). Most importantly, when and under what conditions the earliest familiarity-related neural signals can be detected during perceptual familiarization remains unknown. Addressing this question has implications beyond theoretical interests extending to applied domains. For instance, in forensics, validated measures to objectively determine the level of familiarity an eyewitness could have acquired via a brief encounter or to gauge the amount of learning required for accurate person recognition.

### Measuring visual familiarity neurally

Because successful recognition is a primary behavioral consequence of familiarity, most neural studies have relied on explicit recognition paradigms. However, findings on the neural basis of familiarity have been inconsistent despite clear behavioral changes following familiarization. Neuroimaging studies have reported mixed results of core face-selective regions, including the occipital face area (OFA) and fusiform face area (FFA). Some studies show reduced responses to visually familiar relative to unfamiliar faces (Leveroni et al., 2000; Kosaka et al., 2003; Gobbini and Haxby, 2006), whereas others report increased activation (Katanoda et al., 2000; Apps and Tsakiris, 2014). Importantly, studies have reported that while there are no significant differences using univariate approaches, multivariate analyses reveal distinct voxel activation patterns for visually familiar versus unfamiliar faces in the OFA and FFA (Natu and O’Toole, 2011; Ramon et al., 2015), which emerge prior to explicit behavioral recognition responses (Hahn and O’Toole, 2017). Together, these findings suggest that a neural familiarity signal is largely implicit and does not always correspond to explicit recognition. Consistent with this dissociation, studies of prosopagnosia reported physiological indices of familiarity in the absence of overt recognition (Bruyer et al., 1983; Bauer, 1984).

Unlike recognition-based measures, visual familiarity should be less susceptible to higher cognitive and potentially interfering processes (Hahn et al., 2016). Therefore, directly and objectively measuring the earliest neural signals of visual familiarity requires a paradigm that can induce incidental familiarity rapidly across repeated exposures while simultaneously indexing familiarity strength implicitly as a function of accumulated identity information, independent of explicit identity-related decisions. Such a paradigm would make it possible to determine the brain's minimum exposure requirements and the input conditions necessary for the formation of identity representations. To this end, we developed a novel fast periodic visual stimulation electroencephalography (FPVS-EEG) paradigm to capture implicit familiarity as identity learning unfolds with accumulated identity information.

### Measuring implicit cognitive processes via FPVS-EEG

FPVS-EEG has emerged as an effective paradigm for measuring implicit cognitive processes [for a review, see Rossion et al. (2020)]. The paradigm is a variant of frequency tagging approaches originally developed by Regan (1966), in which visual stimuli are presented in rapid, rhythmic sequences designed to exploit the brain’s tendency to synchronize its activity with periodic visual input. This synchronization produces steady-state visual evoked potentials (SSVEP), that is, sustained neural responses at the frequency of stimulation and its harmonics (Regan, 1966; Norcia et al., 2015). A standard FPVS paradigm involves the rapid presentation of base images, such as objects, at a fixed frequency, for example, 6 Hz, with oddball images, such as faces, periodically interspersed at a distinct frequency, for example, every sixth image, resulting in a 1 Hz oddball frequency (Jacques et al., 2020; Yan and Rossion, 2020; Yan et al., 2023). This rapid stimulus presentation limits extended processing of individual stimuli, thereby promoting automatic neural processing and capturing implicit neural signatures related to oddball detection among base stimuli. Critically, FPVS yields high signal-to-noise (SNR) ratios in brief recording periods due to precise stimulus synchronization. Furthermore, because FPVS-EEG does not require overt behavioral responses, with participants typically performing an unrelated orthogonal task, it effectively isolates neural signals from confounding decision-making factors.

Within face perception research, FPVS-EEG has provided objective neural markers of face-related subprocesses, including face categorization (Rossion et al., 2015), face discrimination under varying visual conditions (Liu-Shuang et al., 2014; Dzhelyova and Rossion, 2014a; Rossion et al., 2020), and detection of highly familiar faces under various viewing conditions using naturalistic images (Zimmermann and Rossion, 2017; Campbell et al., 2020; Yan and Rossion, 2020; Yan et al., 2023). It has also proven effective for investigating automatic and implicit visual processing in both healthy and neurologically impaired individuals across diverse populations, including acquired prosopagnosia (Liu-Shuang et al., 2016), developmental dyslexia (Gigleux et al., 2025), and autism (Van der Donck et al., 2019; Qiao et al., 2022). Beyond face processing, FPVS-EEG has been applied in clinical contexts to study implicit semantic memory in healthy aging (Milton et al., 2020) and neurodegenerative disorders (Stothart et al., 2021, 2023).

### Measuring implicit familiarity using FPVS-EEG

With respect to familiarity, previous studies have demonstrated that FPVS can elicit robust and highly selective neural responses to familiar identities embedded within streams of unfamiliar faces, reflecting the activation of established identity representations (Zimmermann and Rossion, 2017; Verosky et al., 2020; Yan and Rossion, 2020; Yan et al., 2023). Crucially, the magnitude of this identity-selective response scales systematically with the degree of familiarity. For example, own faces evoke the strongest responses, followed by personally familiar faces, whereas unfamiliar faces elicit the weakest responses (Campbell et al., 2020). Consistent with this graded pattern, identity-selective responses have been shown to increase following brief, deliberate familiarization procedures in the laboratory (Verosky et al., 2020) and to strengthen further with extended real-world exposure, such as during the development of new social relationships (Campbell and Tanaka, 2021). Together, these findings demonstrate that FPVS is sensitive to progressive changes in identity representations and can track the emergence and strengthening of familiarity as learning unfolds. However, existing FPVS studies of familiarity have primarily focused on paradigms involving deliberate instruction and extended exposure. As a result, they have not yet addressed how identity-selective neural responses emerge at the earliest stages of learning, when unfamiliar faces begin to form a measurable identity trace through brief passive exposure. Consequently, fundamental questions remain unanswered regarding how quickly the brain can establish facial representations that signal familiarity and which types of visual input are sufficient to support such learning.

To address these questions, we propose a novel paradigm designed to measure neural identity learning across varied visual input conditions within an FPVS-EEG framework. The paradigm presents streams of unfamiliar face images at a base frequency of 6 Hz while periodically inserting an oddball target face at a frequency of 1 Hz. Two critical sequences are employed. In the identity learning sequence, the same oddball identity repeatedly appears among changing base identities, allowing identity-specific familiarization to occur. In an image-type matched control sequence, different oddball identities alternate with changing base identities, providing a measure of general face-identity discrimination without familiarity-based learning. Crucially, participants remained naive as to the learning manipulation and received no explicit encoding instructions, thereby modeling the incidental and passive learning processes characteristic of real-world face-identity acquisition. Stimuli were drawn from the Surveillance Cameras Face Database (SCface; Grgic et al., 2011), which includes both mugshot-quality and CCTV-style images. This made it possible to systematically examine how suboptimal image conditions influence implicit neural learning signals, providing insights into the robustness of unfamiliar face learning under realistic forensic conditions. By linking passive exposure with objective neural indices of familiarization, the present paradigm addresses critical gaps in our understanding of implicit familiarity formation and offers a novel methodological foundation for both theoretical investigation and applied forensic contexts.

## Materials and Methods

### Code accessibility

The raw data and analysis code described in this paper are freely available online at osf.io/3gxu4. The analyses were run on a desktop PC with Windows 11.

### Power analysis

Because this is the first FPVS paradigm to measure implicit familiarity as perceptual, i.e., exposure-related learning unfolds, no directly comparable prior effect sizes are available. Accordingly, the priori estimate was grounded in the closest FPVS face-learning studies. In Campbell and Tanaka’s (2021) work, the identity-specific oddball response increased from pre- to postfamiliarization yielding a medium-to-large learning effect (*N* = 9; *d* = 0.74). Such an effect represents an upper bound, as the familiarization procedure involved long-term, real-life social interaction (2 months). Conversely, Verosky et al. (2020) reported a smaller familiarity effect (*N* = 25; *d* = 0.42) after minimal laboratory familiarization (12 less-familiar faces viewed for ∼9 s each vs 12 more-familiar faces viewed for ∼19 s each). Because Verosky et al. involved many identities, short exposure, and delayed testing, this effect serves as a lower bound. The current paradigm assesses within-session online learning via repeated exposure to a single oddball identity, and the expected effect therefore lies between these bounds. For this reason, an effect size between *d* = 0.50 and *d* = 0.60 was adopted for the comparison between the Learning condition (learning from repeated oddball targets) and the Control condition (no learning due to changing oddball identities). A paired-sample power analysis (*α* = 0.05, two-tailed, 80% power) based on this range indicates a required minimum sample size of 24 participants.

### Participants

Our sample consisted of 28 participants, 16 undergraduate students tested in a controlled laboratory environment (14 females; mean age, 28 ± 8 years) and 12 police officers tested at their workplace (four females; mean age, 37 ± 8 years), all with normal or corrected vision, and no reported neurological or psychiatric history. The procedures complied with the ethical guidelines and data protection regulations of the University of Malta and University of Lausanne. Prior to EEG testing, participants completed the computerized Yearbook Test (YBT; Bruck et al., 1991) and the extended version of the Cambridge Face Memory Test (CFMT+; Russell et al., 2009) to assess face-processing ability. The scores of all participants fell within normal ranges (YBT, 9 ± 4; CFMT+, 67 ± 10), i.e., no participants qualified as Super-Recognizers (SRs) according to the diagnostic framework for lab-based SR identification proposed by Ramon (2021).

We included two distinct samples tested in different environments to assess the robustness and generalizability of the FPVS paradigm across populations and recording contexts rather than draw conclusions about group differences in FIP. The two groups differed substantially in demographic composition, with the civilian sample primarily consisting of young adult females and the law enforcement sample predominantly comprising of middle-aged males. Testing environments also differed, with civilians assessed in a laboratory-based Faraday cage and law enforcement officers tested at their workplace. These differences introduce multiple potential confounds, including age, gender, and signal quality, which preclude straightforward interpretation of group-level neural differences. Accordingly, no direct comparisons between groups are reported.

### Stimuli

#### Identity selection

All images were sourced from the SCface database, a publicly available dataset developed for research on face recognition and verification under real-world, surveillance-like conditions (Grgic et al., 2011). We selected a total of 87 Caucasian male-appearing identities (due to the limited number of female-appearing identities in the database). Individuals with visually distinctive features, such as unusual facial structure, skin tone, prominent facial hair, or eyewear, were excluded to minimize attentional bias or incidental familiarization caused by salient visual cues. We selected three identities that were similar in general appearance (age, hair and eye color, no distinctive facial features or outfit, etc.) to serve as the targets to probe implicit learning.

#### Image selection and preprocessing

For each identity, we extracted two sets of five images: (1) mugshot images, five high-resolution mugshot images captured with a professional camera from five viewpoints rotating around the face, and (2) CCTV, five low-resolution surveillance-like images captured by five distinct CCTV cameras with varying image characteristics, mounted 2.6 m overhead to simulate individuals approaching a surveillance point (for more technical details, see Grgic et al., 2011). All images were processed to ensure consistent facial region size and position. First, face regions were detected using the Pyfacer library (FacePerceiver, 2022), which incorporates the RetinaFace model for face localization (Deng et al., 2020) and the FaRL model for face parsing/segmentation (Zheng et al., 2022). The face region occupied ∼80% of the image region, with the image center adjusted to approximately fall just below the nose. All images were resized to 250 × 250 pixels (96 dpi, 24 bit color depth), yielding a visual angle of ∼4.09 × 4.09° when viewed from 70 cm. While image resizing preserved the clarity of high-resolution mugshot images, the lower resolution of the original CCTV images resulted in visible pixelation. This intentional degradation preserved the ecological validity of the surveillance-like conditions reflected in real-world forensic materials.

#### FPVS paradigm

**Figure 1.**
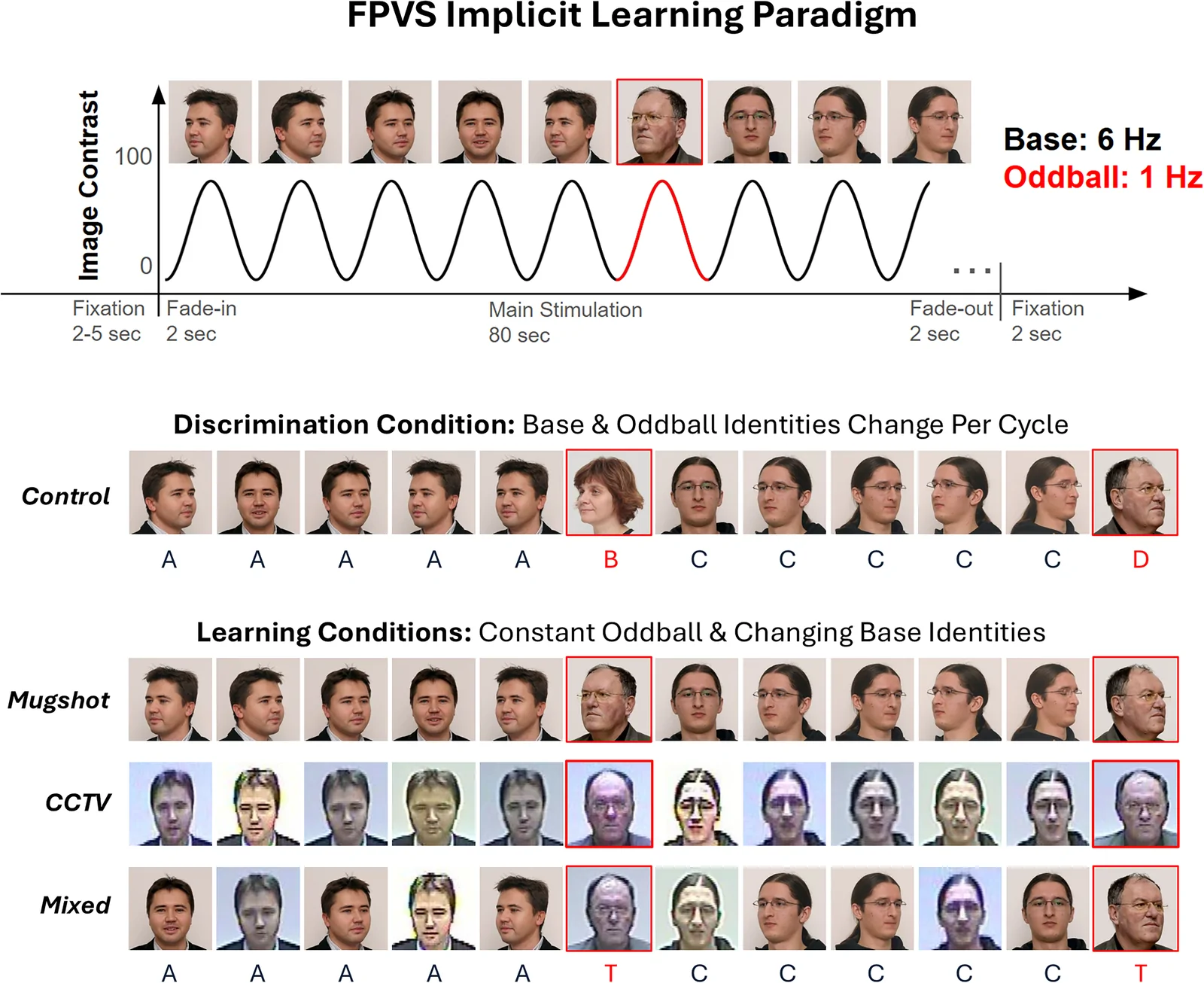
Illustration of the experiment paradigm. Note that only four representative identities are shown to adhere to image copyright agreement.

Our FPVS paradigm (Fig. 1) involved a total of four conditions: a Control condition using only mugshot images designed to measure face discrimination and three Learning conditions using different types of images: Mugshots only, CCTV-like images only, and Mixed both mugshots and CCTV. All 87 identities were used in the Control condition. For the three Learning conditions, we selected three target identities and randomly assigned them to each condition. The remaining 84 identities were split into three unique sets of 28 identities serving as base identities for each condition, as described below.

#### Control condition

The Control condition exclusively used mugshot images from the full 87-identity pool. A trial sequence contained base and oddball identities changing across 84 1 s cycles (e.g., a cycle, AAAAAB; a sequence, AAAAAB CCCCCD; …; base frequency 6 Hz, oddball frequency 1 Hz) to probe face discrimination at every cycle. The identities were pseudorandomly assigned as base or oddball identities so that no single identity was repeated in consecutive base or oddball cycles. Additionally, the three target identities never appeared as base identities. There were four trials in the Control condition.

#### Learning conditions: Mugshot, CCTV, and Mixed

In the Mugshot condition, all five mugshot images (different viewpoints) of each identity were used; in the CCTV condition, all five CCTV-like images (from different cameras) of each identity were used; and in the Mixed condition, a combination of a frontal mugshot and two slightly left or right viewpoint images and two CCTV images from two different cameras for each identity was used.

We probed implicit learning using a sequence of face images flickered at 6 Hz, with each cycle consisting of five images of the same base identity followed by an oddball image of the target identity. While base identities changed across cycles, the oddball identity remained constant throughout the entire trial (a cycle, AAAAAT; a sequence, AAAAAT BBBBBT; …), tagging learning-related activity at 1 Hz. Each Learning condition involved four trials, with each trial composed of three consecutive segments. Each segment contained 28 cycles, corresponding to 28 unique base identities. This resulted in a total stimulation duration of 84 s (3 segments × 28 cycles). The order of base identities was randomized within each segment, and the order of images for each base identity was randomized within each cycle. Oddball images were pseudorandomly drawn from a set of five condition-specific images to prevent image repetition between two consecutive cycles.

#### Visual stimulation

All face stimuli were presented through a sinusoidal duty cycle (from 0 to 100% and back down to 0% within an image cycle of ∼167 ms). Each sequence lasted 84 s, including a 2 s fade-in at the beginning and a 2 s fade-out at the end, during which contrast modulation gradually ramped up from 0 to 100% and down from 100 to 0%, respectively. The total 16 trials (4 conditions × 4 trials) were split into four blocks, with each block consisting of one trial from each condition. The trial order was randomized within blocks.

#### Task and procedure

The experiment took ∼20–25 min to complete (including breaks, excluding EEG cap preparation). During each trial, participants performed an orthogonal task by monitoring and responding to the color change of a central fixation point (from black to red, nonperiodic, 500 ms). They were instructed to press the spacebar when they saw that the fixation cross turned red. This ensured attention to the display without directing explicit focus to the identity learning task. We also recorded two additional conditions to assess face/house category selectivity, replicating Rossion et al. (2015). The data of these conditions were not used in this study.

### Apparatus and EEG recording

For the student group, data collection took place at the Cognitive Science Laboratory at the University of Malta. Participants were seated in a specialized Faraday cage designed for optimal EEG recording conditions. This electromagnetically shielded enclosure effectively isolated EEG measurements from external electrical interference by blocking ambient electromagnetic noise from power lines, electronic devices, and radio frequencies. The facility also provided acoustic isolation to further enhance recording quality. To maintain the integrity of the electromagnetic shielding, participants were explicitly instructed not to bring any electronic devices into the cage during the experimental sessions. For the police officer group, we performed the recordings at their workplace (St Gallen Cantonal Police, Switzerland). Participants were prepared, instructed, and tested in a quiet room. During testing all electronic devices including routers in the room and the neighboring rooms were switched off. Participants were instructed to switch their devices off or use airplane mode. The same computer and monitor were used for the two groups.

Stimuli were presented on a Samsung SyncMaster 2233RZ-3D LCD monitor with an average luminance of ∼110 cd/m² and a resolution of 1,680 × 1,050 pixels. The monitor operated at a 60 Hz refresh rate and was connected to an NVIDIA GeForce GTX 1660 graphics card. All visual stimuli were displayed against a neutral gray background. A custom software application specifically designed for FPVS-EEG experimentation and kindly provided by Bruno Rossion was used to deliver a stimulus presentation. EEG data were acquired using a 64-channel Biosemi ActiveTwo system with Ag/AgCl active electrodes arranged according to the extended 10–20 international system and sampled at 4,096 Hz. The recording setup included four additional external electrodes for monitoring eye movements, along with the system's common mode sense and driven right leg electrodes that serve as the recording reference and ground, respectively. Two HEOG electrodes were placed 1 cm lateral to the external canthi and aligned with the pupils to detect horizontal eye movements. For vertical eye movements, two VEOG electrodes were positioned in vertical alignment above and below with the right pupil. Impedance was kept below 20 kΩ in most participants (when not possible in one participants, impedance was kept under 40 kΩ).

### Data analysis

EEG data were preprocessed using the open-source software Letswave7 (https://github.com/NOCIONS/Letswave7). Cluster-permutation statistics were implemented using the Fieldtrip Toolbox (v20181231; Oostenveld et al., 2011). Both Letswave7 and Fieldtrip Toolbox were executed in MATLAB R2022b (The MathWorks Inc., 2022). Statistical analyses were conducted in RStudio v4.4.1 (R Core Team, 2024). Statistical assumptions of homoscedasticity, sphericity in the repeated-measures design, and normality of variable distribution were statistically tested using Levene's test for homogeneity of variance, Mauchly's test, and the Shapiro–Wilk test, respectively. When violations of these assumptions were detected, appropriate corrections were applied. For sphericity violations, Greenhouse–Geisser corrections were implemented when the estimated epsilon was below 0.75, and Huynh–Feldt corrections were applied when the estimated epsilon exceeded this threshold. Finally, Bonferroni’s corrections were applied for multiple comparisons when applicable (all *p* values reported are corrected). To address random sample variability, we used a nonparametric percentile bootstrap (10,000 resamples with replacement) to estimate the sampling distribution and reliability of the effect (Rousselet et al., 2023).

#### EEG preprocessing

Continuous raw EEG data were first notch filtered to remove line noise at 50 Hz (notch width 2 Hz, slope width 2 Hz), then bandpass filtered at 0.5–100 Hz using a fourth order zero-phase Butterworth filter. Data were then downsampled to 1,024 Hz to reduce computational load. The EOG electrodes were discarded, and eyeblink artifacts were corrected using an independent component analysis (ICA) procedure. Full continuous data were downsampled to 256 Hz to reduce computational load during ICA. The computed ICA weights were then applied to the original 1,024 Hz data. Only components clearly representing eyeblink artifacts were manually identified and removed. Bad channels were manually identified during visual inspection; only two participants required interpolation of one bad channel using three surrounding electrodes. Finally, the EEG sequences were rereferenced to the common average of all 64 electrodes. Preprocessed continuous EEG data were segmented into individual trials starting from 2 s after fade-in until right before 2 s before fade-out period, including the exact 80 cycles of oddball cycles (1 Hz). Individual trial segments were transformed into the frequency domain using fast Fourier transform applied to extract amplitude spectra for each participant, with a frequency resolution of 0.0125 Hz (calculated as 1/80 s).

#### Harmonics analysis

For each condition, grand-average amplitude spectra were calculated across participants, trials, and channels. A *z*-score transform was applied on the condition grand-averaged spectra by subtracting the amplitude at each frequency of interest to the averaged amplitude of surrounding frequency bins within a 0.10 Hz margin (7 bins on each side, excluding the immediately adjacent bins) and then divided by the standard deviation of the surrounding bins. Significant responses were considered at a threshold of *z* > 3.1 (*p* < 0.001, one-tailed, signal > noise). We identified significant consecutive harmonics in any condition separately for Base frequency *F* *+* 6 Hz and Oddball frequency *f* *+* 1 Hz.

#### Individual SNR magnitude

For each participant, amplitude spectra were averaged across four trials within each condition. SNR was calculated by dividing the amplitude at the frequency of interest and the mean amplitude of 14 surrounding frequency bins within 0.10 Hz, excluding the next immediate bins. Base responses *F+* were calculated as the summation of SNR at significant Base harmonic frequencies *F+* (6, 12, 18, and 24 Hz; see Results), averaged across 64 channels. Individual oddball responses were calculated as the summation of SNR at the Oddball significant harmonic frequencies *f+* (1, 2, 3, 4, and 5 Hz; see Results), averaged across 64 channels.

#### Implicit learning analysis (Control vs Mugshot)

Our first analysis sought to determine whether implicit learning of oddball identity could be measured by comparing oddball responses *f+* between Control and Mugshot conditions, using paired-sample *t* tests. We also compared base responses *F+* to test whether a different oddball structure would lead to differences in base responses. We further conducted a cluster-permutation analysis using individual average trial SNR with a full frequency range from 1 to 30 Hz. This analysis was agnostic to frequency types (Base or Oddball), aiming to identify any significant clusters grouped by frequency neighbors and spatial proximity between the two conditions (clusters' *t* statistics calculated using *maxsum* function; two-tailed cluster alpha of 0.01 with 5,000 permutations).

#### Image-type effect analysis (Mugshot vs CCTV vs Mixed)

We tested the effect of image types on neural responses in the three Learning conditions, for base and oddball responses separately, using repeated-measures ANOVAs.

#### Implicit learning at individual levels

To determine the presence of significant oddball responses in each condition, for each participant, we calculated amplitude spectra averaged across the four trials using 64 channels and 11 electrodes in the occipital–temporal (OT) regions of interest (ROIs) identified in previous studies (P7, P9, PO7, O1, Iz, Oz, POz, P8, P10, PO8, O; Rossion et al., 2020; Yan et al., 2023). We then computed a *z*-score based on the summed amplitude at the oddball frequency and its significant harmonics (1–5 Hz), using a local noise estimate derived from the surrounding 14 frequency bins (±0.10 Hz range, excluding the immediately adjacent bins). To determine individual significant learning responses (Mugshot > Control), we subtracted individual amplitude spectrum of the Control from the Mugshot and then applied the same *z*-scoring procedure to the differential spectrum, using the enhanced signals observed at harmonics 2–3 Hz (based on cluster-permutation analysis; see Results below). A *z*-score above 1.64 (*p* < 0.05, one-tailed, signal > noise) was considered statistically significant (Yan and Rossion, 2020).

## Results

### Significant frequency and harmonics analysis

**Figure 2.**
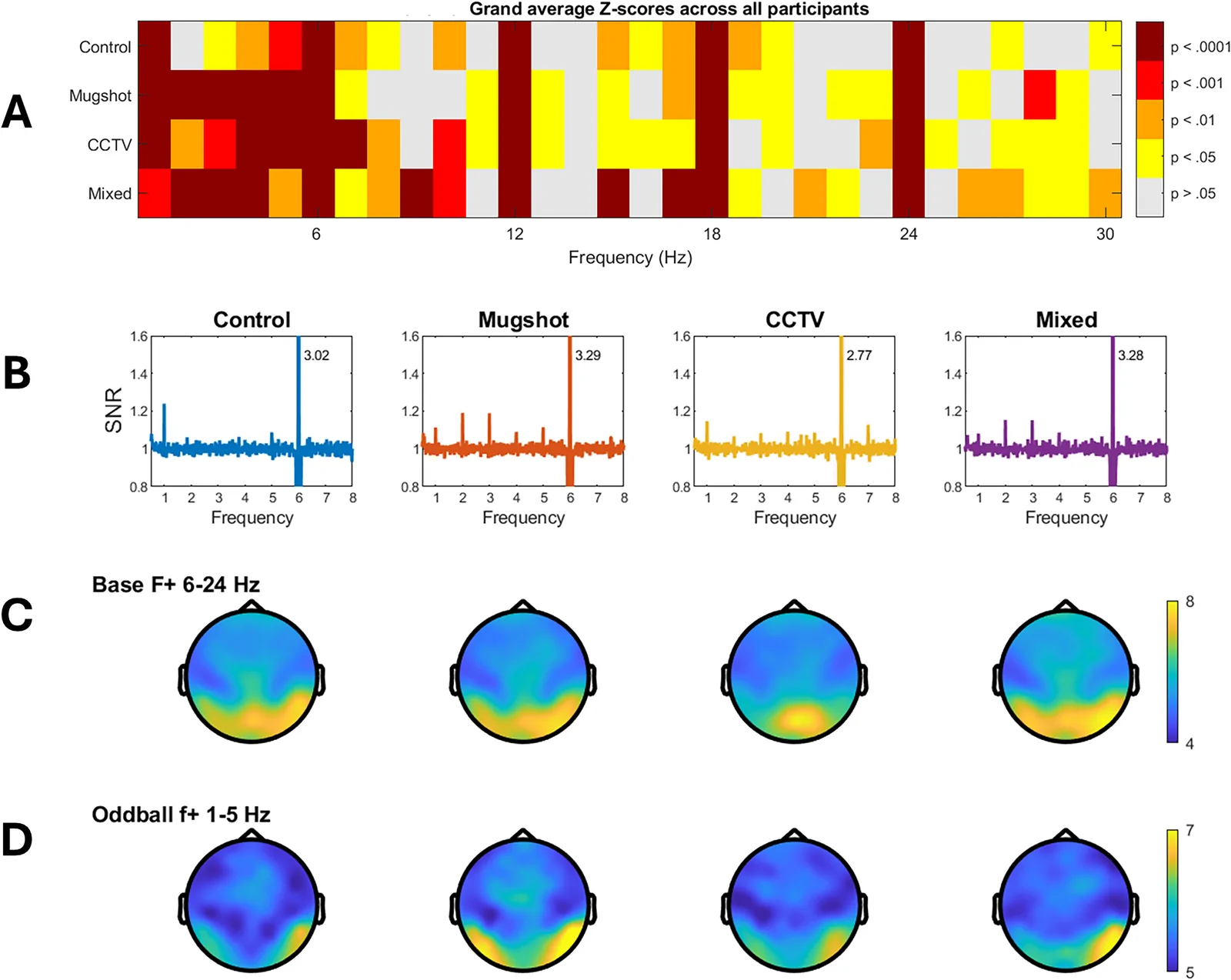
***A***, Group-averaged *z*-score across four conditions at integer frequencies in range from 1 to 30 Hz. ***B***, Grand-averaged SNR. ***C***, ***D***, Scalp plots for base responses *F* *+* 6–24 Hz and oddball responses *f* *+* 1–5 Hz.

At base frequency *F+*, responses were significant (*z* > 3.1; *p* < 0.001) at the fundamental base frequency of 6 up to 24 Hz (Fig. 2*A*) predominantly at lateral OT regions (Fig. 2*C*). Thus, we quantified base response *F+* as the sum of amplitude at these significant harmonic frequencies (6, 12, 18, and 24 Hz). For response at oddball frequency 1 Hz, significant oddball responses were found in consecutive harmonic frequencies up to 5 Hz (Fig. 2*A*), predominantly in lateral OT regions (Fig. 2*D*). Oddball responses were thus quantified as the summed responses of 1, 2, 3, 4, and 5 Hz.

### Implicit learning: Control versus Mugshot

#### Group-level analysis

**Figure 3.**
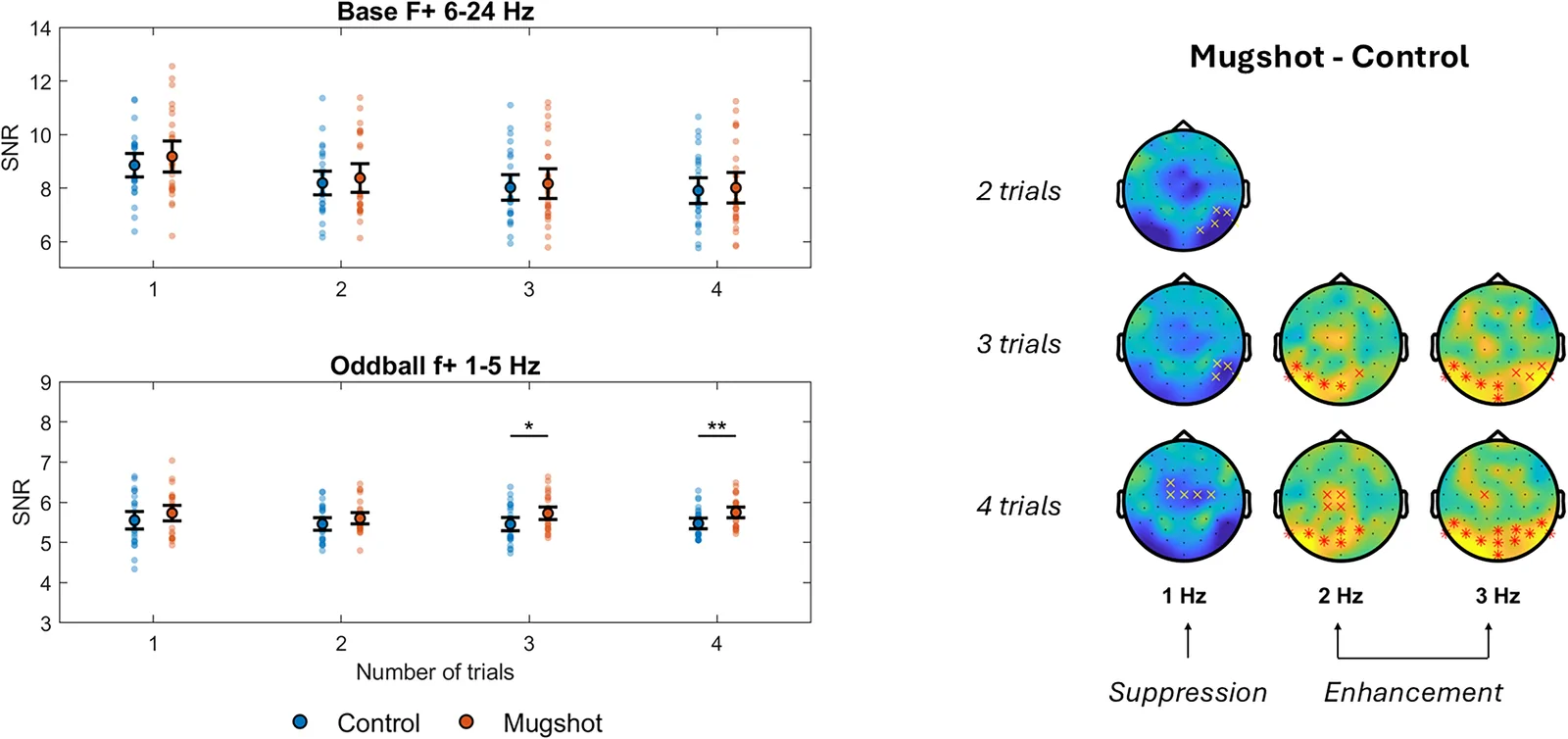
Control and Mugshot comparisons. The left panel shows SNR responses as a function of number trials for Base *F* *+* 6–24 Hz (top) and Oddball *f* *+* 1–5 Hz (bottom). The right panel shows cluster-permutation two-tailed–dependent significant statistical results as a function of the number of trials considered (yellow markers indicate reduced amplitudes, Mugshot < Control; red markers indicate response increased amplitudes, Mugshot > Control).

Our first critical question was whether the proposed FPVS paradigm could probe implicit learning of repeated oddball identity, controlling for varying oddball identities (Fig. 3, left panel). To address this question, our main statistical test used SNR of amplitude spectra averaged across four trials and 64 channels, summing across significant oddball frequency and harmonics identified earlier, i.e., Oddball *f* *+* 1–5 Hz. A paired-sample *t* test comparing Control and Mugshot conditions showed a significant difference, with SNR magnitude in the Control condition (*M* = 5.47; SE = 0.07) being significantly lower than in Mugshot condition (*M* = 5.75; SE = 0.07; *t*_(27)_ = −3.36; *p* = 0.008; 95% CI [−0.43, −0.11], a moderate to high effect; Cohen's *d* = 0.64). The nonparametric bootstrap on the paired differences using 10,000 resamples with replacement yielded a 95% bootstrap CI of [−0.43, −0.12] (*p* = 0.0004), indicating a robust difference between Control and Mugshot conditions (Mdiff = 0.28).

To roughly explore the time-course of this effect over four blocks of trials, we conducted additional comparisons using cumulative data from the first trial, the first two trials, and the first three trials. We found that the learning effect emerged at the third block (Bonferroni corrected *p* = 0.048; bootstrap *p* = 0.003 and 95% CI [−0.47, −0.08]). For responses at Base *F* *+* 6−24 Hz, there were no significant differences between Control and Mugshot conditions, regardless of the number of trials considered (Table 1).

**Table 1.** Control–Mugshot: statistical results

Trials	Control	Mugshot	_*t*(27)_	*p*	Cohen's *d*	95% CI
Base *F* *+* *6–30 Hz*						
1	8.85 (0.22)	9.18 (0.30)	−1.71	*0*.*099*	−0.32	[−0.70, 0.05]
2	8.19 (0.23)	8.38 (0.27)	−1.17	*0*.*253*	−0.22	[−0.50, 0.13]
3	8.02 (0.24)	8.17 (0.28)	−0.97	*0*.*340*	−0.18	[−0.43, 0.15]
4	7.91 (0.24)	8.01 (0.29)	−0.61	*0*.*549*	−0.11	[−0.46, 0.24]
Oddball *f* *+* *1–5 Hz*						
1	5.55 (0.11)	5.73 (0.10)	−1.32	*0*.*198*	−0.25	[−0.44, 0.09]
2	5.46 (0.08)	5.60 (0.07)	−1.38	*0*.*177*	−0.26	[−0.34, 0.06]
3	5.45 (0.08)	5.72 (0.08)	−2.70	*0*.012*	−0.51	[−0.47, −0.07]
4	5.47 (0.07)	5.75 (0.07)	−3.36	*0*.002**	−0.64	[−0.43, −0.11]

*p < 0.05; **p < 0.01.

#### Cluster-permutation analysis

Post hoc cluster-permutation two-tailed tests between Control and Mugshot conditions were conducted across the full frequency range of 1–30 Hz, using one- to four-trial aggregated SNR amplitudes. This enables a trial-based temporal analysis of learning patterns accumulated over trials. Overall, significant differences emerged exclusively at lower frequencies, specifically at 1, 2, and 3 Hz (Fig. 3, right panel), predominantly over the OT regions consistent with previous FPVS face studies (Rossion et al., 2020; Yan et al., 2023).

#### Temporal progression of learning signatures

Analysis of the temporal progression revealed systematic changes across trial aggregations. At the second trial, a 1 Hz signal reduction (Mugshot < Control) was observed over the right OT (rOT) regions. At the third trial, this 1 Hz reduction over rOT persisted, while an enhancement pattern (*Mugshot *> *Control*) was observed at 2 Hz over left OT (lOT) regions and at 3 Hz over bilateral OT regions. At the fourth trial, the 1 Hz reduction concentrated at central-midline electrodes, while enhancement patterns were most pronounced at 2–3 Hz over bilateral OT as well as central-midline channels.

#### Individual-level significant response

**Figure 4.**
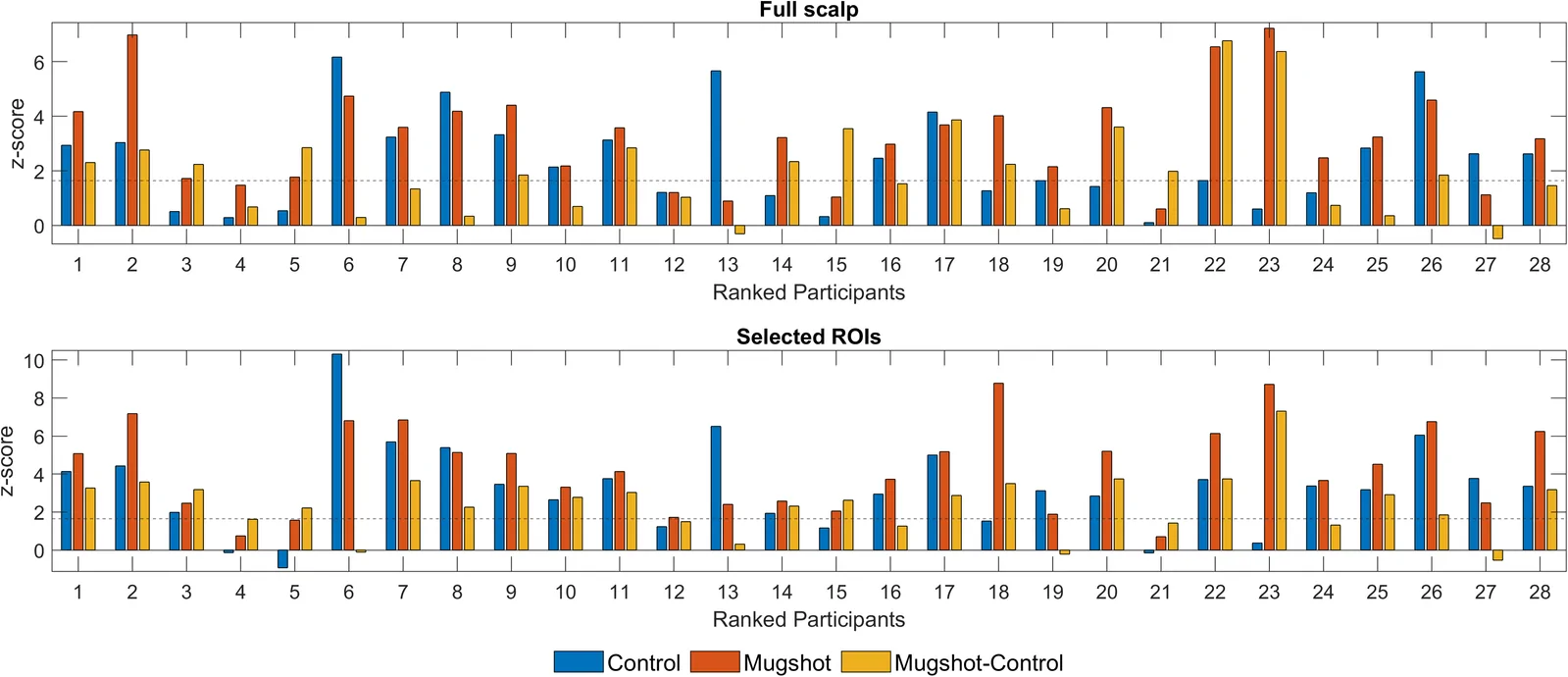
Individual oddball responses at 1–5 Hz for Control and Mugshot and 2–3 Hz for differential Mugshot–Control amplitude. Horizontal dash line at *z*-cutoff of 1.64 (*p* < 0.05). Participants were ranked by a combined normalized score (YBT and CFMT+) in an ascending order.

The individual oddball responses at significant harmonic frequencies 1–5 Hz (quantified as *z*-score > 1.64; *p* < 0.05, one-tailed; Yan and Rossion, 2020) were significant in 17 of 28 participants in the Control condition and increased to 22 out of 28 participants in the Mugshot condition (Fig. 4). Implicit learning using differential responses between Mugshot and Control found significant learning responses in 15 participants. We repeated the same analysis but restricted it to posterior ROIs including electrodes over lateral OT areas identified in the earlier cluster-permutation analysis. We observed that the number of significant responses increased: 21 participants in Control, 25 in Mugshot, and 19 in Mugshot–Control (Fig. 4). However, we found no significant correlations between these neural responses and participants' behavioral scores on the CFMT+ or YBT.

#### Low-level image statistics analysis

A substantial body of work has shown that early visual processing is strongly constrained by the statistical regularities of natural images. Natural scenes exhibit characteristic distributions of luminance, contrast, and spatial frequency content, most notably a scale-invariant 1/*f* structure, and early visual neurons are well matched to these statistics (Field, 1987). Neural representations and perceptual sensitivity are adapted to these regularities, such that deviations from typical natural image statistics can lead to atypical sensory responses and perceptual performance (Simoncelli and Olshausen, 2001; Geisler, 2008). Accordingly, verifying that stimuli fall within the typical range of natural image statistics reduces the likelihood that observed neural differences are driven by low-level sensory factors, although such controls do not eliminate all possible confounds.

To assess whether the Mugshot–Control difference could be explained by low-level pictorial properties specific to the target identity, we quantified global image statistics for all stimuli in the Mugshot condition using the exact images presented during the experiment. Metrics were computed for five images per identity and averaged to obtain identity-level summaries. For each image (250 × 250 pixels), we computed mean luminance (grayscale mean), RMS contrast (grayscale standard deviation), spatial frequency content quantified as the spectral slope of the radially averaged Fourier amplitude spectrum (log–log slope, excluding the DC component), and mean color hue in HSV space after excluding low-saturation pixels (saturation > 0.10).

The target identity was evaluated relative to the nontarget identity pool using percentile ranks and a permutation-based outlier test. Percentiles were computed as the proportion of nontarget identities with values lower than the target. Outlier probabilities were estimated using a two-sided permutation test relative to the pooled median, following robust statistical recommendations (Rousselet et al., 2023). Specifically, the absolute deviation of the target identity from the median of the pooled identity distribution was compared against a null distribution generated by randomly resampling (with replacement) a single identity value from the pooled set (20,000 permutations; fixed random seed).

**Figure 5.**
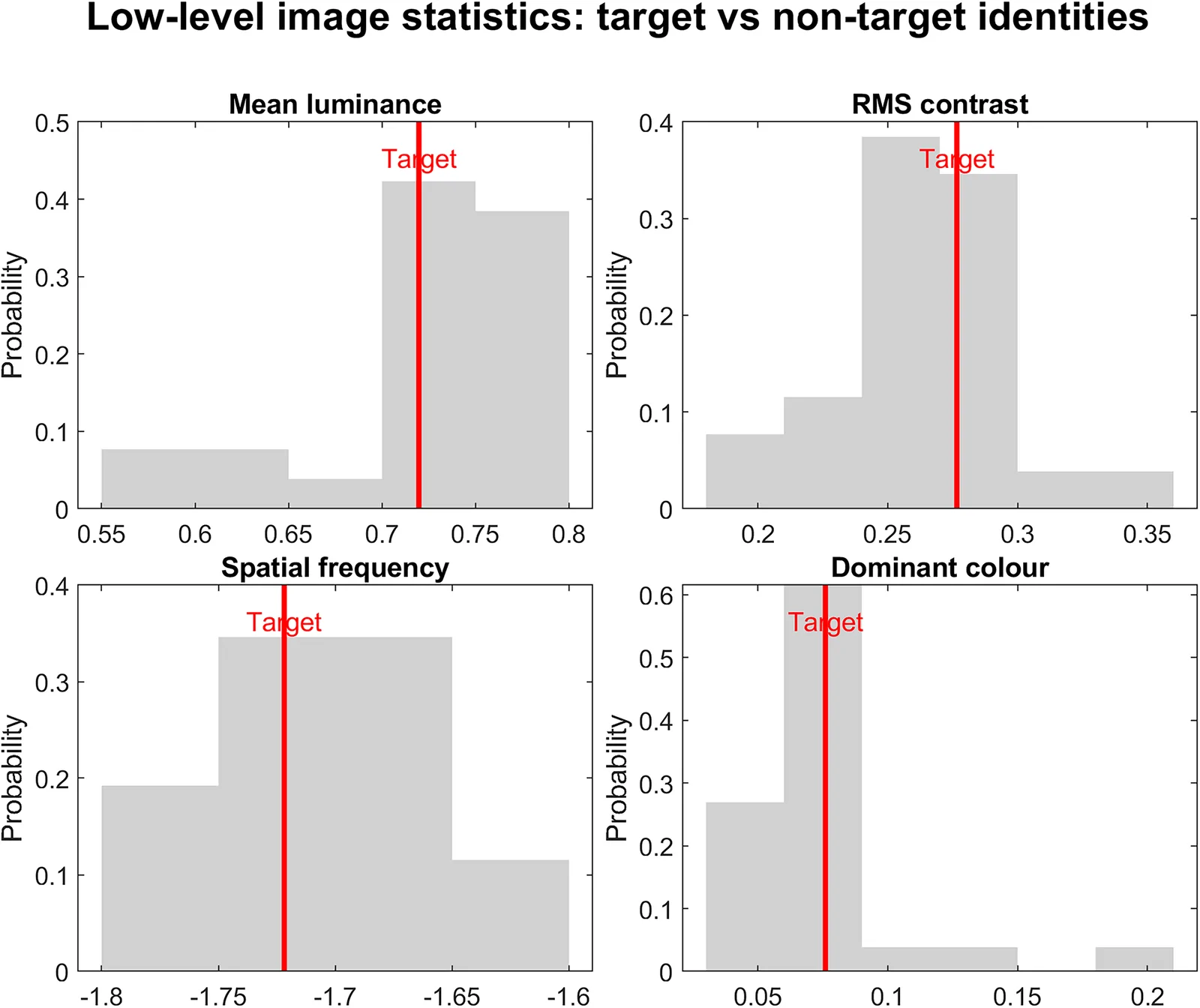
Comparison of the target identity with the distribution of nontarget identities across low-level image properties in the Mugshot condition.

Across all metrics as shown in Figure 5, the target identity fell well within the distribution of nontarget identities. Mean luminance of the target (0.72) closely matched the nontarget distribution (*M* = 0.72; SD = 0.06; *p* = 0.630). RMS contrast for the target (0.28) was slightly higher than the nontarget mean (*M* = 0.26; SD = 0.03) but remained within the central range of the distribution (*p* = 0.550). Spatial frequency content showed comparable values for the target (spectral slope = −1.72) and nontarget identities (*M* = −1.70; SD = 0.05; *p* = 0.710). Mean color hue for the target (0.08) was also typical relative to nontarget identities (*M* = 0.08; SD = 0.03; *p* = 0.630). Across all measures, permutation-based outlier tests indicated no significant deviation of the target identity from the nontarget identity pool (all *p* > 0.55), reducing the likelihood that the Mugshot–Control difference is driven by low-level pictorial properties of the target image set.

### Effect of image type: Mugshot–CCTV–Mixed

#### Group-level analysis

**Figure 6.**
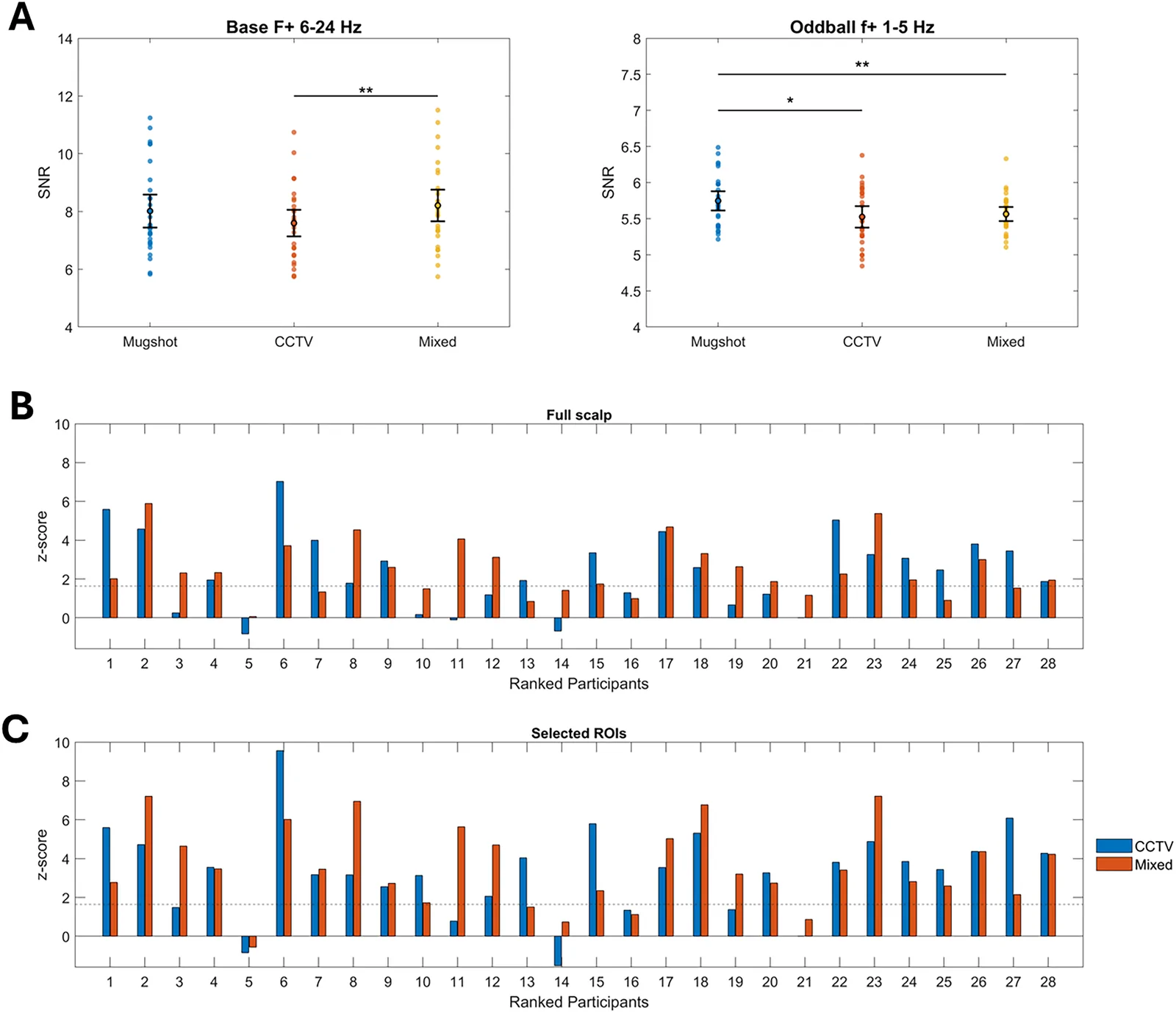
***A***, Group-average SNR across three Learning conditions for base responses and oddball responses. ***B***, ***C***, Individual oddball responses *f* *+* 1–5 Hz using averaged 64-channel amplitude and averaged ROIs, respectively, with the horizontal dash line indicating the *z*-cutoff value at 1.64 (*p* *<* 0.05). Participants were ranked by a combined normalized score (YBT and CFMT+) in an ascending order.

Comparing Oddball *f* *+* 1–5 Hz among the three Learning conditions, ANOVA test showed a significant effect of Condition (*F*_(2,54)_ = 6.43; *p* = 0.006; *η*^2^ = 0.075), indicating a medium effect size (Fig. 6*A*). Post hoc tests showed that responses in the Mugshot condition (*M* = 5.75; SE = 0.07) were significantly higher than those in the CCTV condition (*M* = 5.52; SE = 0.08; *t*_(27)_ = 3.28; *p* = 0.012; 95%CI [0.08, 0.36], Cohen's *d* = 0.58) and responses in the Mugshot condition were also significantly higher than those in the Mixed condition (*M* = 5.56; SE = 0.05; *t*_(27)_ = 3.52; *p* = 0.008; 95%CI [0.08, 0.29]; Cohen's *d* = 0.58). There was no difference between CCTV and Mixed conditions (*t*_(27)_ = 0.51; uncorrected *p* = 0.613; 95%CI [−0.12, 0.20]; Cohen's *d* = 0.12).

For base response *F* *+* 6–24 Hz, we found a significant effect of Condition (*F*_(2,54)_ = 6.16; *p* = 0.004; *η*^2 ^= 0.032; Fig. 6*A*). Post hoc analysis indicated that base response in the Mixed condition (*M* = 8.21; SE = 0.28) was significantly higher than in the CCTV condition (*M* = 7.60; SE = 0.23; *t*_(27)_ = 3.54; *p* = 0.004; 95%CI [0.26, 0.97]; Cohen's *d* = 0.45). For the remaining comparisons with Mugshot condition (*M* = 8.01; SE = 0.29), no significant differences were found (Mixed–Mugshot, *t*_(27)_ = 1.02; uncorrected *p* = 0.317; Mugshot–CCTV, *t*_(27)_ = 2.45; *p* = 0.084).

#### Individual-level significant response

We found significant oddball responses in 18 participants in the CCTV condition and 19 participants in the Mixed condition using 64-channel *z*-transformed amplitude (Fig. 6*B*). When neural responses were restricted to OT ROIs, the number of participants showing significant responses increased to 21 in CCTV and 23 in Mixed condition (Fig. 6*C*). We found no significant correlations between these neural responses and participants' behavioral scores on the CFMT+ or YBT.

## Discussion

There is a substantial body of research demonstrating familiarity advantages in FIP (Hancock et al., 2000; Johnston and Edmonds, 2009; Ramon and Gobbini, 2018), where familiar face processing shows remarkable robustness across visual variations (Bruce and Young, 1986; Jenkins et al., 2011). While ample knowledge about unfamiliar/familiar category boundaries exists (Ramon et al., 2011; Caharel et al., 2014; Barragan-Jason et al., 2015; Besson et al., 2017; Huang et al., 2017), the neural processes by which unfamiliar faces become familiar remains poorly understood, particularly when and under what conditions the earliest familiarity-related neural signals can be detected during perceptual familiarization (cf. Kovács, 2020).

We proposed a novel FPVS-EEG paradigm to objectively assess the emergence of implicit familiarity through repeated passive visual exposure. Our FPVS paradigm employs a frequency tagged oddball design in which a single oddball identity appears at 1 Hz within a 6 Hz stream of changing base identities. This design enables implicit familiarity to be indexed by comparing neural responses elicited by a repeatedly presented oddball identity with oddball responses elicited by changing identities. To examine how image quality influences implicit familiarity signals, we used both mugshot-quality and CCTV-style images, allowing systematic assessment of familiarity formation under ecologically valid input conditions that closely mirror those encountered in forensic contexts.

Our findings demonstrate that neurotypical observers with average FIP abilities exhibit robust neural markers of implicit familiarity during passive face repetition learning. Crucially, implicit familiarity emerged within just four minutes of rapid visual stimulation when high-quality facial images were used, observed in 68% of participants. This neural marker of implicit familiarity was characterized by two partially overlapping neural patterns, namely, suppression at 1 Hz and enhancement at 2–3 Hz, each associated with a distinct spatial distribution that is described in detail below. Importantly, optimal viewing conditions proved critical for the emergence of early implicit familiarity, indicating that within the fixed exposure duration used here, a minimum level of image quality is required for facial familiarization to become objectively detectable. We discuss these findings and their broader theoretical and applied implications below.

### Neural characteristics of implicit familiarity

Our findings indicate that neural markers of implicit familiarity emerging during identity learning are characterized by two concurrent patterns. First, suppression was observed at the fundamental oddball frequency of 1 Hz over rOT regions. This pattern is consistent with repetition suppression effects previously reported for repeated presentation of a single identity and likely reflects generic repetition-related mechanisms (Nemrodov et al., 2015). Second, enhancement was observed at higher oddball harmonics, specifically 2–3 Hz, over bilateral OT regions with possible additional central-midline involvement. This enhancement is consistent with prior FPVS studies reporting increased responses to familiar—relative to unfamiliar—faces following familiarization (Verosky et al., 2020; Campbell and Tanaka, 2021).

The co-occurrence of suppression and enhancement involved overlapping rOT regions, consistent with previous FPVS studies of face processing (Rossion et al., 2020) and with intracerebral recording studies that have localized face-selective responses along the lateral inferior occipital gyrus and lateral fusiform gyrus (Jacques et al., 2020; Hagen et al., 2025). Such localization also overlaps with lesion sites associated with acquired prosopagnosia (Meadows, 1974; Sergent and Signoret, 1992; Bouvier and Engel, 2006; Barton, 2025), supporting the functional relevance of these effects for FIP. Together, the present findings suggest that repetition suppression and familiarity enhancement can coexist within the same neural system, potentially explaining mixed neuroimaging results in core face-selective regions, with reports of both repetition suppression (Leveroni et al., 2000; Kosaka et al., 2003; Gobbini and Haxby, 2006) and increased activation (Katanoda et al., 2000; Apps and Tsakiris, 2014) following familiarization.

The neural synchronization model proposed by Gotts et al. (2012) offers a potential account for how these patterns may coexist. According to this framework, repetition can reduce overall firing rates through mechanisms such as neural fatigue, sharpening, or facilitation, giving rise to suppression at the fundamental frequency (Grill-Spector et al., 2006), or through reduced prediction error as top–down expectations are formed for repeated stimuli (Summerfield et al., 2008; Grotheer and Kovács, 2016). At the same time, repetition may increase the temporal precision of the remaining spikes, leading to enhanced stimulus sensitivity associated with repetition priming (Grill-Spector et al., 2006). Such synchronization may contribute to increased responses at higher harmonics observed in the Learning condition compared with the Control condition here.

### Harmonic components in FPVS oddball responses

The present findings raise important questions regarding the origin and functional significance of harmonic components in FPVS and, more broadly, in SSVEP studies of perception and memory. Steady-state responses are known to contain energy at harmonic frequencies because neural responses are not purely sinusoidal and because both stimulus structure and neural processing introduce nonlinearities (Regan, 1966; Norcia et al., 2015). In FPVS and SSVEP paradigms, harmonic components are therefore a common and expected feature of the measured response. In oddball FPVS specifically, the periodic insertion of oddball stimuli corresponds to a nonsinusoidal temporal modulation with an unequal duty cycle, which necessarily generates harmonic structure in the frequency domain, with nonlinear neural processing further contributing to harmonic energy (Liu-Shuang et al., 2014; Norcia et al., 2015).

Importantly, prior FPVS work has typically treated responses at the oddball frequency and its harmonics as components of the same periodic discrimination response rather than as evidence for distinct neural or cognitive mechanisms, motivating the standard practice of combining harmonic responses to improve measurement sensitivity and reliability (Liu-Shuang et al., 2014; Retter et al., 2021). At the same time, evidence from the broader SSVEP literature indicates that harmonic components can be differentially modulated by task demands such as attention, with effects sometimes observed at higher harmonics but not at the fundamental frequency (Mahajan et al., 2022). However, previous literature and the present paradigm do not assert an interpretation of the observed harmonic differences as indexing distinct neural mechanisms (see Discussion in Rossion et al., 2015).

Rather, the pattern of concurrent suppression and enhancement across frequencies is considered informative. Previous SSVEP research in repetition priming and memory has reported similar findings within the same experimental context (Radtke et al., 2021). Furthermore, enhancement of cortical activity has been linked to explicit retrieval processes and recognition memory (Voss and Paller, 2008; Martens et al., 2012a, b). To our knowledge, no other FPVS studies have reported systematic modulation of the harmonic profile of the oddball response following learning. In this respect, the present findings provide a novel observation, suggesting that harmonic components may contain additional information about how identity representations evolve with repeated exposure, even though the precise physiological significance of individual harmonics remains to be clarified. Clarifying how harmonic distributions relate to identity learning and face-specific processing therefore represents an important direction for future research in FPVS paradigms.

### The role of image quality

The comparison between mugshot-quality and CCTV-like images revealed that optimal viewing conditions are critical for detecting reliable implicit familiarity in our neurotypical observers. In other words, image degradation sharply reduces the neural signatures associated with identity learning, in line with previous behavioral studies that show high error rates when matching CCTV materials to photographs (Bruce et al., 2001) or to live targets (Davis and Valentine, 2009). Our paradigm can at the very least offer a partial solution to this problem by supplying an objective neural criterion. Specifically, our results suggest minimum image quality thresholds for a specific exposure duration (high-quality mugshots viewed ∼4 s), below which familiarization of unfamiliar identities after brief exposures becomes empirically unsupportable for observers with average FIP abilities.

Importantly, without image-matched controls for CCTV and Mixed conditions, we cannot definitively establish whether identity learning is completely absent or merely reduced when using degraded materials. The relationship between image quality and exposure duration may involve trade-offs that require further investigation. Furthermore, the reduced neural responses we observed with CCTV materials may reflect the inherent limitations of typical face-processing abilities in our participant sample. However, previous research suggests that individuals with exceptional face recognition skills (i.e., SRs) can extract meaningful information from images that fall below standard quality thresholds (Ramon, 2021). We are currently investigating this possibility in ongoing work.

### Brain–behavior relationships

We found no significant correlations between neural responses and performance on the CFMT+ or the YBT. Previous studies have reported inconsistent relationships between implicit and explicit face-processing tasks using FPVS-EEG, with no correlation in Yan et al. (2023), a weak correlation with CFMT scores in Xu et al. (2017), and a strong correlation with the Benton Face Recognition Test in Dzhelyova et al. (2020). The lack of correlation in our study likely stems from multiple factors. Firstly, detecting a correlation of *r* = 0.30 reported in Xu et al. (2017; *N* = 49) with 80% power would require ∼64 participants. With a sample size of 28 participants, the present study was underpowered to detect small to moderate neural–behavioral associations, limiting strong inferences at the individual-differences level. Additionally, EEG signals recorded at the scalp are influenced by individual differences in skull thickness, brain-to-scalp distance, cortical folding, and source orientation, all of which affect signal amplitude independently of neural processing efficiency (Ahlfors et al., 2010; Jacques et al., 2020; Yan et al., 2023), introducing variability that can obscure relationships with behavioral performance in smaller samples.

Beyond statistical considerations, the absence of neural behavioral correlations may reflect a more fundamental conceptual dissociation between what the current FPVS task measures and what behavioral tests assess. Dissociations between implicit familiarity and explicit recognition have been reported previously, both in neurotypical observers and in clinical populations (Bruyer et al., 1983; Bauer, 1984; Hahn et al., 2016; Hahn and O’Toole, 2017; Ambrus et al., 2021, p. 201). From this perspective, FPVS responses in the present study index the emergence of familiarity-related neural responses prior to and partially dissociated from explicit recognition measures in behavioral tasks.

More broadly, the difficulty of identifying straightforward neural behavioral relationships in face recognition likely reflects the inherent complexity of the face-processing network, as well as the highly varied (and imperfect) behavioral metrics subject to investigation (cf. Fysh et al., 2020; Bobak et al., 2023). Substantial individual variability in both the structure and function of this network has been documented in neurotypical individuals across development (Scherf et al., 2007). Even populations often assumed to be relatively homogeneous in face-processing ability exhibit marked diversity in their neural and behavioral profiles, including so-called SRs (Belanova et al., 2018; Ramon, 2021; Faghel-Soubeyrand et al., 2024) and individuals with congenital prosopagnosia (Manippa et al., 2023). This pronounced heterogeneity suggests that univariate neural measures may be insufficient to capture the nuanced differences underlying face-processing abilities, while multivariate approaches might prove more sensitive to reveal meaningful brain–behavior relationships (Elbich and Scherf, 2017; Genon et al., 2022).

### General methodological considerations

An anonymous reviewer raised the concern that the detection of periodic identity changes or repeated identity learning, in principle, could have been aided by reliance on external cues such as hairstyle or clothing present in our stimuli. Although the contribution of external features cannot be fully ruled out, we contend that our reported familiarity signals are unlikely to be primarily explained by differences in external features. First, previous research suggests external features do not dominate identity learning when using full-face stimuli (Veres-Injac and Schwaninger, 2009; Kramer et al., 2018a). Second, repeated exposure to whole-face images primarily strengthens representations of internal facial features, even when external cues are available (Longmore et al., 2015). Third, directing attention toward internal features reduces reliance on external information (Fletcher et al., 2008). Consistent with this literature, several aspects of the present paradigm are likely biased processing toward internal facial features, including the requirement for central fixation, the predominance of internal features (80% of total image area) within the stimulus, and the rapid presentation rate (167 ms) which limits peripheral visual exploration.

Another point raised by the reviewer concerns the extent to which low-level image properties specific to the target identity may have contributed to the observed learning-related response. Although low-level image statistics can influence repetition-based learning, we believe that our deliberate curation of the target identities and stimulus set (see Materials and Methods) substantially minimized such contributions. Moreover, prior FPVS research has shown that identity-selective oddball responses are strongly reduced by face inversion and abolished by phase scrambling, indicating a critical dependence on face-specific information rather than on low-level image statistics alone (Liu-Shuang et al., 2014; Dzhelyova and Rossion, 2014b; see Norcia et al., 2015). Importantly, Dzhelyova and Rossion (2014b) demonstrated that isolated manipulations of facial shape or surface information produce markedly weaker responses than when both cues are combined, suggesting that identity-selective neural responses are supra-additive and reflect higher-level face representations.

To further address potential confounds related to luminance, contrast, or spatial frequency content (Field, 1987; Simoncelli and Olshausen, 2001; Geisler, 2008), we conducted a complementary analysis of low-level image statistics. This analysis indicated that the target identity was not a systematic outlier relative to the nontarget identity pool in terms of mean luminance, RMS contrast, spatial frequency distribution, or color hue. These results reduce the likelihood that the learning effect observed here was driven by idiosyncratic low-level pictorial properties of the target image set. Nevertheless, future studies could address this issue more directly through controlled image-based manipulations, such as inversion or phase scrambling, to further dissociate the contributions of low-level image statistics from higher-level identity representations.

### Future directions

An important direction for future work concerns the temporal emergence and evolution of implicit familiarity and its relationship to explicit recognition. At present, it remains unclear whether participants would be able to explicitly recognize the oddball identity if queried directly, leaving a key interpretive gap. Previous studies indicate that subsequently remembered targets elicit larger neural amplitudes than forgotten targets, suggesting that early neural sensitivity may foreshadow later behavioral outcomes (Peterson et al., 2014; Killebrew et al., 2018). Future studies could extend the current paradigm to clarify several key issues: At what point does neural learning become behaviorally accessible? How does implicit familiarity relate to explicit recognition for specific identities and general face-processing ability measured with standardized behavioral tests?

These questions have direct implications on individual differences in implicit face-identity learning. Individuals with exceptional abilities, such as SRs, can extract diagnostic identity information even under brief and degraded viewing conditions (Ramon, 2021; Ramon and Rjosk, 2022), suggesting that the use of degraded inputs can be utilized as a stringent learning threshold well suited for identifying high-performing individuals. Our paradigm can also offer a valuable tool for studying developmental and acquired prosopagnosia, where explicit recognition is impaired despite partially preserved implicit processing (Bruyer et al., 1983; Bauer, 1984; Tranel and Damasio, 1985; Eimer et al., 2012). Future adaptation of our paradigm involving systematic variation of image quality, learning duration, and recognition phase could clarify whether superior or impaired performance reflects differences in early perceptual encoding or in the consolidation of identity representations for later recognition.

### Conclusion

This study introduces a novel FPVS-EEG paradigm to objectively capture the emergence of implicit familiarity responses during passive visual exposure to facial identities. By embedding systematic identity repetition within a rapid visual stream, the paradigm isolates identity-specific learning signals without requiring explicit recognition or task engagement. Using ecologically valid stimuli that varied in image quality enabled assessment of implicit familiarity formation under viewing conditions that approximate real-world identification contexts. Our results show that implicit familiarity-related neural signatures can emerge rapidly, within minutes of passive exposure, provided that visual input quality is sufficient. Critically, degraded visual input markedly attenuate learning-related neural responses in neurotypical observers, indicating minimum visual input requirements for identity learning within a fixed exposure window. This sensitivity makes the paradigm potentially well suited for probing individual differences in face-processing capacity, particularly in high performers (Nador and Ramon, 2021; Ficco and Ramon, 2025; Nador et al., 2025; Ramon, in press).

Beyond its theoretical contributions, our FPVS-EEG approach provides a rapid and quantifiable measure of implicit familiarity that reduces exposure to decision-making and cognitive biases inherent in recognition-based judgments (Dror, 2020). With further replication and generalization across identities and samples, our novel paradigm could support the development of neural benchmarks across graded levels of image quality, helping to delineate conditions under which identity learning is neurally detectable following brief or degraded exposure. Such benchmarks may inform future work on personnel screening in roles requiring face-learning expertise (Nador et al., 2022; Mayer and Ramon, 2023) and contribute to ongoing discussions about the reliability and interpretability of behavioral tests in high-stakes identification contexts (Bobak et al., 2023).

## Synthesis

Reviewing Editor: Anne Keitel, University of Dundee Decisions are customarily a result of the Reviewing Editor and the peer reviewers coming together and discussing their recommendations until a consensus is reached. When revisions are invited, a fact-based synthesis statement explaining their decision and outlining what is needed to prepare a revision will be listed below. The following reviewer(s) agreed to reveal their identity: NONE.

The revised manuscript "Neural Face Identity Learning via Passive Exposure and FPVS-EEG" has now been assessed by the editor, as none of the two original reviewers were available. One reviewer refused to review the revision when they realised that no new experiments had been added, and the other agreed to review, but did not submit a review, and did not respond to numerous attempts to reach them. The editor deemed that responses to concerns had all been answered well.

For future responses to reviewers, it is strongly recommended to treat the reply as a stand-alone document, which includes quotes of changed/added text from the manuscript, including line numbers, and new/changed figures, under each reply where relevant. This would be a massive help for reviewers and speed up the review process.

Congratulations on the acceptance of your manuscript! All the best, Anne
